# National Burden of Bicyclist Hospitalizations in the United States by Rurality, 2016–2020

**DOI:** 10.1111/jrh.70194

**Published:** 2026-07-28

**Authors:** Amir Ghanbari, Ryan M. Carnahan, Joseph E. Cavanaugh, Jodie M. Plumert, Elizabeth E. O'Neal, Cara Hamann

**Affiliations:** ^1^ Department of Epidemiology University of Iowa Iowa City Iowa USA; ^2^ Injury Prevention Research Center University of Iowa Iowa City Iowa USA; ^3^ Department of Biostatistics University of Iowa College of Public Health Iowa City Iowa USA; ^4^ Department of Psychological and Brain Sciences The University of Iowa College of Liberal Arts and Sciences Iowa City Iowa USA; ^5^ Department of Occupational and Environmental Health University of Iowa Iowa City Iowa USA

**Keywords:** bicyclist injuries, healthcare systems, hospitalization, injury, rurality

## Abstract

**Purpose:**

Bicyclist injuries are a major public health concern in the United States, with limited understanding of variations in injury severity and discharge outcomes by rurality. This study provides a national assessment of bicycling‐related hospitalizations, focusing on differences by rurality.

**Methods:**

Using HCUP‐NIS data (2016–2020), bicycling‐related hospitalizations were identified. Descriptive analyses characterized demographic and regional trends. Multivariable logistic regression, stratified by rural/urban patient residence, was used to assess factors associated with non‐routine discharges.

**Findings:**

Between 2016 and 2020, an estimated 124,000 bicyclist‐related hospitalizations occurred, resulting in more than $10 billion in hospital costs. Overall, 31.2% resulted in non‐routine discharge, including 1.4% deaths. Hospitalizations were stable through 2019 but increased by >20% in 2020. Urban residents accounted for most cases (91.1%). Cases were predominantly male (80%); aged 31–70 (65.4%) and hospitalized in the South (31.2%) or West (36.4%). Increasing age showed a strong dose–response relationship with non‐routine discharge in both settings (e.g., age ≥ 71: OR_rural_ = 15.71, 95% confidence interval [CI]: 9.52–25.93; OR_urban_ = 12.18, 95% CI: 10.21–14.52). Motor‐vehicle involvement increased the odds of non‐routine discharge in both settings (OR_rural_ = 2.53, 95% CI: 1.89–3.40; OR_urban_ = 1.68, 95% CI: 1.55–1.82).

**Conclusions:**

Bicyclist hospitalizations persisted and slightly increased over the study period. Age was the strongest predictor of non‐routine discharge across settings, whereas sex and income effects were significantly associated with outcomes in urban areas. Findings highlight the need for tailored prevention strategies and equitable infrastructure improvements across rural and urban settings.

## 1 Introduction

Bicycling has long been recognized as a sustainable and health‐promoting mode of transportation that offers cardiovascular benefits, reduces all‐cause mortality, and is cost‐effective relative to other modes of travel [1]. However, recent surges in popularity have been accompanied by persistent and often growing safety concerns. The risks of injury and death among bicyclists are disproportionately high compared to those of motor vehicle occupants [2]. Over 130,000 bicyclists are injured and more than 1000 die in crashes each year in the United States [3]. Despite comprising less than 1% of total trips, bicyclists account for more than 2% of traffic‐related fatalities [4].

Over 600,000 bicycle‐related injuries are treated in emergency departments (EDs) annually in the United States [5, 6]. Hospitalization data reveal a more troubling trend: Bicycling‐related hospital admissions have increased significantly in recent decades in both number and severity. A more than doubled increase in adult hospitalizations occurred between 1998 and 2013—from 5.1 to 11.2 per 100,000 people [7]. More recent data from the COVID‐19 era confirm this escalation, with cycling being the only sport to show an increase in ED visits during 2020 [8].

An important component of developing effective prevention strategies is to establish a comprehensive picture of current injury patterns and their associated burden. Several national studies have examined bicycling injuries using ED data, which primarily capture initial treatment encounters and also include patients admitted or transferred for hospitalization. For example, Sanford et al. used the National Electronic Injury Surveillance System (NEISS) to show increases in head trauma, age shifts, and hospital admissions through 2013 [7], whereas Johnson et al. analyzed NEISS data from 2012 to 2021, reporting a decline in overall injuries but an increasing burden among older adults [9]. Although these studies provide valuable national estimates of injury trends, they lack detailed inpatient outcomes such as length of stay (LOS), hospital charges, and discharge disposition. In contrast, hospitalization data are essential for understanding the full scope of serious injuries, healthcare usage, and long‐term outcomes. The most recent nationally representative analysis of bicycling‐related hospitalizations analyzed data from 2002 to 2009 [10]. Nevertheless, since that analysis, there have been major transitions, such as the switch from ICD‐9 to ICD‐10 coding, as well as changes in cycling trends and infrastructure, all of which underscore the need for updated information.

In addition to the type of crash (i.e., single bicycle vs. motor vehicle involved), the geographical setting in which an injury occurs, such as urban versus rural, may also play a critical but underexamined role in hospitalizations. Rural and urban environments differ significantly in terms of road conditions, crash characteristics, and access to trauma care, all of which could affect the severity and outcome of bicycle injuries. Prior research in Canada found that children living in rural areas had substantially higher hospitalization rates for both bicycling‐related head and non‐head injuries, a pattern they attributed to limited proper bicycling infrastructure and lower helmet use rates [11]. Similarly, studies from the United States have reported that patients hospitalized for unintentional injuries, including road traffic crashes, had higher admission rates and incurred greater hospital charges in rural compared with urban settings [12]. Although adjusted analyses show that bicycling prevalence is generally similar across geographic regions [13], studies have also documented that the burden of injury is greater and the capacity to provide timely and appropriate hospital care is more limited in rural areas [12]. Despite evidence of disparities in injury‐related hospitalizations by rurality, national studies have rarely examined how rurality shapes the overall burden of hospitalization. This gap limits our understanding of how rural–urban differences influence injury outcomes and care delivery on the national level. The aim of this study was to examine the distribution and trends in bicycling‐related injury hospitalizations, identify predictors of clinical outcomes, and assess whether these outcomes differ by rurality.

## 2 Methods

### 2.1 Study Design and Data Source

Data for this study were drawn from the National Inpatient Sample (NIS), Healthcare Cost and Utilization Project (HCUP), Agency for Healthcare Research and Quality for the years 2016 to 2020 [14]. The NIS is the largest publicly available all‐payer inpatient healthcare database in the United States, capturing a stratified sample of hospital discharges from community hospitals and representing more than 95% of the US population. To represent all inpatient discharges from US hospitals at a national level, NIS employs a 20% stratified sample of discharges from all participating hospitals, clustering at the hospital level, and discharge‐level sampling weights.

The study population included hospitalized bicyclists identified using ICD‐10‐CM external cause‐of‐injury codes V10–V19, which cover a range of transport‐related incidents involving bicyclists. These include collisions with motor vehicles, non‐motor vehicles, pedestrians, stationary objects, and other non‐collision events. To maximize case capture, codes were searched across the first four external cause fields (I10_ECAUSE1 through I10_ECAUSE4). Moreover, the study period was selected to align with ICD‐10‐CM adoption, ensuring uniform classification across years.

### 2.2 Study Variables

#### 2.2.1 Outcome Variable

The primary outcome variable was discharging disposition, categorized as routine (discharge to home) and non‐routine (in‐hospital death, transfer to another hospital or care facility, discharge against medical advice).

#### 2.2.2 Exposure Variables

The primary exposure variable was patient residential location, treated as an effect modifier for the other covariates of interest. Due to lack of geographic injury location data in the NIS, this variable was used as proxy for crash location. This approach is supported by a growing body of research indicating that traffic‐related injuries frequently occur close to home. For instance, over 70% of motor vehicle crashes (MVCs) have been shown to occur within 10 mi of the driver's residence [15]. Likewise, the median distance from home to crash site is reported as 4.6 mi for car or motorcycle crashes, compared to just 1.2 mi (IQR: 0.4–2.7) for injured cyclists [16]. Expanding on this evidence, another study found that non‐motorists, including cyclists, are more likely to crash within their own ZIP code, likely due to limited travel range [17]. This pattern was further confirmed through geospatial analyses indicating that up to 92% of bicycle crashes occurred within the same ZIP code as the cyclist's residence [18]. These consistent findings across multiple studies support the use of patient residence as a suitable proxy for crash location in bicycling injury research. Rurality was determined on the basis of the National Center for Health Statistics (NCHS) classification, with counties in areas of fewer than 50,000 population designated as rural [19].

Covariates examined in this study included a broad range of patients, hospitals, and injury characteristics, which were selected on the basis of previous literature and the data availability of the NIS.

#### 2.2.3 Patient Demographics and Socioeconomic Factors

Demographic variables included age (0–9, 10–19, 20–30, 31–50, 51–70, and 71+ years); sex (male or female); and race/ethnicity as provided in NIS, which codes this as a single, mutually exclusive variable. Categories in NIS include White, Black, Hispanic, Asian or Pacific Islander, Native American, and other/unknown. For analysis, the smaller categories (Asian or Pacific Islander, Native American, and missing) were combined into a single “other/unknown” group to ensure adequate sample sizes and stable estimates. Socioeconomic status was approximated using the median household income quartile of the patient's 5‐digit ZIP code, ranging from Quartile 1 (lowest 0–25th percentile) to Quartile 4 (highest 76–100th percentile). The primary payer for hospitalization was recorded as Medicare/Medicaid, private insurance, self‐pay/no charge, or other/unknown.

#### 2.2.4 Hospital and Injury‐Related Measures

Hospital and injury characteristics were incorporated to contextualize the observed disparities in bicyclist injury burden. Hospital bed size (small, medium, or large) served as a proxy for institutional capacity, whereas geographic region (Northeast, Midwest, South, or West) captured regional differences. ED admission status indicated whether patients were first treated in the ED prior to hospitalization. Transfer‐in status, categorized as direct admission, transfer from another acute‐care hospital, or transfer from another healthcare facility, reflected variations in access to definitive care.

To examine crash context, two additional factors were included: motor vehicle involvement, identified using ICD‐10‐CM codes V12–V15, and admission timing, categorized as weekday or weekend, which served as a proxy for differing trip purposes (e.g., commuting vs. recreational cycling). Injury severity was quantified using the New Injury Severity Score (NISS) and Revised Injury Severity Score (RISS), both derived from ICD‐10‐CM codes using the Association for the Advancement of Automotive Medicine (AAAM) ICD‐ISS tool (v2.0). NISS enhances sensitivity by summing the squares of the three most severe injuries regardless of body region, whereas RISS applies reverse weighting to minimize the impact of outliers, addressing the skewed distributions often seen in administrative datasets [20].

### 2.3 Analysis

Distributions of patient and hospital characteristics over the study period (2016–2020) were analyzed using descriptive statistics. To examine geographic location as a possible effect modifier, the analyses were stratified by the residence of patients (urban versus rural). To calculate national estimates, all analyses incorporated the NIS survey design and discharge weights. A full model, including all main effects and interaction terms between patient residence and other covariates, was tested to formally assess effect modification. For urban and rural subgroups, separate multivariable logistic regression models were formulated to examine relationships between exposure variables and discharge disposition. A manual stepwise selection approach was used on the basis of Bayesian Information Criterion (BIC), as BIC is more conservative and generally performs better than the Akaike Information Criterion (AIC) for descriptive modeling in large sample applications [21]. All analyses were conducted using SAS version 9.4.

## 3 Results

### 3.1 Descriptive Characteristics of Hospitalized Bicyclists

#### 3.1.1 Hospitalization Costs, Lengths of Stay, and Discharge Disposition

Between 2016 and 2020, an estimated 124,000 inpatient hospitalizations for bicycle‐related injuries occurred in the United States (Table 1). These injuries translated into a substantial financial burden, with total hospitalization costs exceeding $10 billion dollars during the 5‐year period. Of this amount, approximately $734 million was attributed to rural patients, whereas the remainder stemmed from urban patients. On average, the national hospital LOS was 4.90 days (95% CI: 4.79–5.02). LOS was similar between rural (4.94 days, 95% CI: 4.59–5.29) and urban patients (4.85 days, 95% CI: 4.73–4.97). However, the average total hospital charges differed substantially, with urban hospitalizations averaging $85k (95% CI: $83k–$88k) per person compared to $67k (95% CI: $62k–$72k) in rural settings. Nationally, the overall mean hospital charge per person was $85k (95% CI: $82k–$87k). Time trends also showed that the total number of hospitalizations remained relatively stable from 2016 to 2019 but increased from 24,000 in 2019 to 29,675 in 2020, representing a 23.6% increase. In terms of discharge outcomes, 31.2% of hospitalizations resulted in non‐routine discharge, which included 1.4% who died during the hospital stay. The proportions of discharge dispositions were similar across rural and urban settings.

**TABLE 1:** Characteristics of bicyclist injury hospitalizations by rurality^a^, National Inpatient Sample (NIS), 2016–2020, United States.

	NIS			National estimates					
				Total		Urban		Rural	
Characteristic	Total	Urban	Rural	*N*	%	*N*	%	*N*	%
**Year**	24,819	22,617	2202	124,095	100	113,085	91.1	11,010	8.9
2016	4792	4388	404	23,960	19.3	21,940	19.4	2020	18.3
2017	4709	4270	439	23,545	19.0	21,350	18.9	2195	19.9
2018	4583	4146	437	22,915	18.5	20,730	18.3	2185	19.8
2019	4800	4330	470	24,000	19.3	21,650	19.1	2350	21.3
2020	5935	5438	452	29,675	23.9	27,415	24.3	2260	20.5
**Patient characteristics**									
**Age**									
0–9	1007	833	174	5035	4.1	4165	3.7	870	7.9
10–19	2466	2157	309	12,330	9.9	10,785	9.5	1545	14.0
20–30	2336	2141	195	11,680	9.4	10,705	9.5	975	8.9
31–50	6172	5724	448	30,860	24.7	28,620	25.3	2240	20.3
51–70	10,098	9266	832	50,490	40.7	46,330	40.9	4160	37.8
≥71	2740	2496	244	13,700	11.0	12,480	11.0	1220	11.1
**Sex**									
Male	19,857	18,165	1692	99,285	80.0	90,825	80.3	8460	76.8
Female	4957	4447	510	24,785	20.0	22,235	19.7	2550	23.2
**Race/Ethnicity**									
White	16,956	15,224	1732	84,780	71.0	76,120	69.8	8660	83.5
Black	2094	1967	127	10,470	8.8	9835	9.0	635	6.1
Hispanic	3061	2953	108	15,305	12.8	14,765	13.5	540	5.2
Other/Unknown	1778	1670	108	8890	7.4	8350	7.7	540	5.2
**Primary insurance payer**									
Medicare/Medicaid	10,918	9815	1103	54,590	44.1	49,075	43.5	5515	50.3
Private insurance	10,796	9978	818	53,980	43.6	49,890	44.2	4090	37.3
Self‐pay/No charge	2169	1973	196	10,845	8.8	9865	8.7	980	8.9
Other/Unknown	887	813	74	4435	3.6	4065	3.6	370	3.4
**Median household income**									
Quartile 1 (lowest)	5780	4956	824	28,900	23.6	24,780	22.1	4120	38.7
Quartile 2	5514	4669	845	27,570	22.5	23,345	20.9	4225	39.7
Quartile 3	6077	5715	362	30,385	24.8	28,575	25.6	1810	17.0
Quartile 4 (highest)	7128	7029	99	35,640	29.1	35,145	31.4	495	4.6
**Hospital and injury characteristics**									
**Discharge disposition**									
Routine	17,056	15,508	1548	85,280	68.8	77,540	68.6	7740	70.3
Non‐routine^b^	7749	7096	653	38,745	31.2	35,480	31.4	3265	29.7
—Transfer to short‐term hospital^c^	3989	3605	384	19,945	16.1	18,025	15.9	1920	17.4
—Home healthcare (HHC)	2892	2684	208	14,460	11.6	13,420	11.9	1040	9.5
—Against medical advice (AMA)	517	480	37	2585	2.1	2400	2.1	185	1.7
—Died	351	327	24	1755	1.4	1635	1.5	120	1.1
**Hospital region**									
Northeast	4026	3763	263	20,130	16.2	18,815	16.6	1315	11.9
Midwest	4008	3368	640	20,040	16.2	16,840	14.9	3200	29.1
South	7745	7034	711	38,725	31.2	35,170	31.1	3555	32.3
West	9040	8452	588	45,200	36.4	42,260	37.3	2940	26.7
**Hospital bed size**									
Small	3292	3062	230	16,460	13.3	15,310	13.5	1150	10.4
Medium	6311	5807	504	31,555	25.4	29,035	25.7	2520	22.9
Large	15,216	13,748	1468	76,080	61.3	68,740	60.8	7340	66.7
**Admission day**									
Weekday	16,957	15,415	1542	84,785	68.3	77,075	68.2	7710	70.0
Weekend	7862	7202	660	39,310	31.7	36,010	31.8	3300	30.0
**Motor vehicle crash (MVC) involvement**									
Non‐MVC	19,653	17,817	1836	98,265	79.2	89,085	78.8	9180	83.4
MVC	5166	4800	366	25,830	20.8	24,000	21.2	1830	16.6
**ED admission**									
No	3975	3509	466	19,875	16.0	17,545	15.5	2330	21.1
Yes	20,844	19,108	1736	104,220	84.0	95,540	84.5	8680	78.9
**Transfer status**									
Not transferred	20,920	19,420	1500	104,600	84.7	97,100	86.3	7500	68.6
Transferred in	3780	3092	688	18,900	15.3	15,460	13.7	3440	31.4
**Injury severity and hospitalization stays**									
NISS, *mean (95% CI)* ^d^	13.8 (13.6–13.9)	13.7 (13.5–13.9)	14.0 (13.4–14.6)						
RISS, *mean (95% CI)* ^d^	10.8 (10.6–10.9)	10.7 (10.6–10.9)	11.1 (10.6–11.6)						
LOS (days), *mean (95% CI)*	4.90 (4.79–5.02)	4.85 (4.73–4.97)	4.94 (4.59–5.29)						
Hospital charges (USD), *mean (95% CI)* ^e^	$85k ($82k–$87k)	$85k ($83k–$88k)	$67k ($62k–$72k)						

*Note*: Chi‐square tests comparing rural and urban patients indicated statistically significant differences for all categorical variables presented in this table (*p* < 0.05), except for admission day and discharge disposition, which did not differ significantly between groups.

Abbreviations: CI, confidence interval; LOS, length of stay; NISS, New Injury Severity Score; RISS, Revised Injury Severity Score.

^a^ Rurality was determined on the basis of patient home address.

^b^ Discharge categories are defined separately in this table for clarity. This differs from subsequent analyses in which discharge disposition was modeled as a binary variable.

^c^ Transfers include discharge to a short‐term hospital, skilled nursing facility (SNF), intermediate care facility (ICF), or another type of facility.

^d^ NISS and RISS values were calculated using unweighted NIS sample data and do not represent national estimates.

^e^ Dollar amounts are rounded to the nearest thousand.

#### 3.1.2 Demographic Characteristics

Older adults aged 51–70 years accounted for the largest proportion of hospitalizations (40.7%), followed by adults aged 31–50 years (24.7%). Overall, males (80.0%) and White patients (71.0%) represented the majority of hospitalized bicyclists. In terms of variation by rurality, children (0–9 years) and adolescents (10–19 years) accounted for a disproportionately greater share of rural hospitalizations compared with urban hospitalizations (7.9% vs. 3.7% and 14.0% vs. 9.5%, respectively). Similarly, White patients comprised a larger proportion of rural hospitalizations (83.5% vs. 69.8%), whereas Black and Hispanic patients accounted for a greater share of urban hospitalizations than rural hospitalizations. Male hospitalizations occurred at comparable rates across rural and urban settings. Additional demographic characteristics are presented in Table 1.

Socioeconomic differences were also apparent, as hospitalized bicyclists from the highest income quartile were more often from urban areas (31.4% urban vs. 4.6% rural), whereas those from the lowest income quartile were more commonly from rural areas (38.7% rural vs. 22.1% urban). Insurance coverage also varied modestly by rurality, with Medicare/Medicaid slightly more prevalent among rural patients (50.3% vs. 43.5%) and private insurance more common in urban areas (44.2% vs. 37.3%).

#### 3.1.3 Hospital and Injury Characteristics

Most hospitalizations occurred in large‐bed hospitals (61.3%), with a higher proportion of admissions to large‐bed facilities among patients from rural areas (66.7%) compared to urban areas (60.8%). Regionally, the West (36.4%) and South (31.2%) accounted for the largest proportions of hospitalizations. Rural patients were more often hospitalized in the Midwest (29.1%) and South (32.3%), whereas urban patients were more commonly hospitalized in the West (37.3%). Most patients (84.0%) were admitted through the ED, with slightly higher ED use in urban settings (84.5%) compared to rural (78.9%). Transfers between hospitals were more frequent among rural patients (31.4%) compared to urban patients (13.7%).

Regarding injury mechanism, less than a quarter of hospitalizations (20.8%) involved a motor vehicle, with non‐MVCs accounting for a greater share of rural than urban hospitalizations (83.4% vs. 78.8%). Hospitalizations occurred more frequently on weekdays (68.3%) than weekends (31.7%), closely reflecting the 5:2 weekday‐to‐weekend ratio, suggesting no notable variation by day of week. Finally, regarding severity of injuries, both NISS and RISS were slightly higher in rural areas (14.0 and 11.1) compared to urban areas (13.7 and 10.7), though differences were minimal.

### 3.2 Multivariable Logistic Regression Modeling

Multivariable logistic regression results for predictors of non‐routine discharge are presented by variable groups (Table 2). Analyses were stratified by rural and urban patient residences, and odds ratios (OR) with 95% confidence intervals (CI) are reported. The analyses identify which demographic, hospital and injury characteristics were associated with non‐routine discharge in each setting. Main effect estimates and interactions with rurality are presented in Table A1 in the Supporting Information Appendix. The results for each variable group are given in the following sections.

**TABLE 2:** Adjusted odds of non‐routine discharge following bicycle‐related hospitalizations, stratified by rurality^a^, National Inpatient Sample (NIS), 2016–2020, United States.

	Rural		Urban	
Variable	OR	95% CI	OR	95% CI
** Patient characteristics**				
**Age**				
10–19 years old	Reference		Reference	
9 and younger	0.48	0.20–1.13	0.44	0.30–0.63
20–30	2.62	1.49–4.60	2.34	1.94–2.82
31–50	4.50	2.84–7.14	3.27	2.78–3.85
51–70	7.37	4.76–11.41	6.50	5.55–7.62
≥71	15.71	9.52–25.93	12.18	10.21–14.52
**Sex** ^b^				
Female	Reference		Reference	
Male	1.10	0.86–1.41	0.81	0.75–0.88
**Race/Ethnicity**				
White	Reference		Reference	
Black	0.91	0.57–1.46	1.01	0.89–1.13
Hispanic	0.70	0.38–1.31	0.83	0.74–0.92
Other/Unknown	1.14	0.66–1.99	0.91	0.80–1.03
**Primary insurance payer**				
Self‐pay	Reference		Reference	
Medicare/Medicaid	3.23	2.03–5.15	2.67	2.33–3.07
Private insurance	1.84	1.14–2.95	1.71	1.49–1.96
Other/Unknown	1.99	0.98–4.05	1.55	1.25–1.92
**Median household income**				
Quartile 1 (lowest)	Reference		Reference	
Quartile 2	1.11	0.87–1.43	1.00	0.91–1.10
Quartile 3	1.05	0.75–1.47	0.93	0.84–1.02
Quartile 4 (highest)	0.88	0.49–1.58	0.88	0.80–0.97
**Hospital and injury characteristics**				
**Hospital region** ^b^				
Midwest	Reference		Reference	
Northeast	1.43	0.98–2.07	1.28	1.14–1.44
South	0.97	0.72–1.30	1.13	1.01–1.25
West	0.63	0.47–0.86	1.03	0.92–1.14
**Admission day**				
Weekend	Reference		Reference	
Weekday	1.22	0.96–1.55	1.01	0.95–1.08
**Transfer status**				
Not transferred	Reference		Reference	
Transferred in	1.17	0.92–1.49	1.16	1.05–1.27
**Motor vehicle crash (MVC) involvement** ^b^				
Non‐MVC	Reference		Reference	
MVC involvement	2.53	1.89–3.40	1.68	1.55–1.82

*Note*: Admission type and hospital bed size were removed from the multivariable models during stepwise selection.

Abbreviation: OR, odds ratio.

^a^ Rurality was determined based on patient home address.

^b^ The interactions between patient residence (urban vs. rural) and sex, hospital region, and motor vehicle crash (MVC) involvement were statistically significant (*p* < 0.05) (see Table A1 in the Supporting Information Appendix).

#### 3.2.1 Demographic Characteristics

Patient age emerged as one of the strongest factors associated with non‐routine discharge that includes in‐hospital death, transfer to another hospital or care facility, and discharge against medical advice in both rural and urban settings, with older age groups having higher odds of non‐routine discharge. Compared to adolescents aged 10–19, older adults 51–70 had higher odds of non‐routine discharge (OR_rural_ = 7.37, 95% CI: 4.76–11.41; OR_urban_ = 6.50, 95% CI: 5.55–7.62), whereas adults aged 71 and older had the highest odds of non‐routine discharge (OR_rural_ = 15.71, 95% CI: 9.52–25.93; OR_urban_ = 12.18, 95% CI: 10.21–14.52). In terms of sex, males had lower odds of non‐routine discharge than females in urban areas (OR_urban_ = 0.81, 95% CI: 0.75–0.88), but this type of association was lacking in rural areas. A similar urban–rural divergence appeared in relation to income with patients in the highest income quartile having lower odds of non‐routine discharge in urban areas (OR_urban_ = 0.88, 95% CI: 0.80–0.97), but similar odds of non‐routine discharge between income groups in rural settings. Comparison of racial and ethnic groups revealed that Hispanic patients in urban areas showed reduced odds of non‐routine discharge compared to White patients (OR_urban_ = 0.83, 95% CI: 0.74–0.92), whereas the odds of non‐routine discharge had negligible racial differences in rural areas. Moreover, all insurance types showed elevated odds of non‐routine discharge compared to self‐pay patients, with the highest odds observed for Medicare/Medicaid (OR_rural_ = 3.23, 95% CI: 2.03–5.15; OR_urban_ = 2.67, 95% CI: 2.33–3.07).

#### 3.2.2 Hospital and Injury Characteristics

Among patients residing in rural areas, those treated in hospitals located in the West had lower odds of non‐routine discharge compared to those treated in the Midwest (OR = 0.63, 95% CI: 0.47–0.86). In contrast, rural patients treated in the Northeast or South did not have substantially different discharge patterns compared to the Midwest. For patients residing in urban areas, treatment in hospitals located in the Northeast (OR = 1.28, 95% CI: 1.14–1.44) and South (OR = 1.13, 95% CI: 1.01–1.25) was associated with higher odds of non‐routine discharge compared to the Midwest. Regarding patient transfer status, those who were transferred to another hospital had higher odds of non‐routine discharge, compared to those not transferred (OR_rural_ = 1.17, 95% CI: 0.92–1.49, OR_urban_ = 1.16, 95% CI: 1.05–1.27). Furthermore, involvement in an MVC also increased the likelihood of non‐routine discharge relative to non‐MVC crashes, with a stronger association in rural areas (OR_rural_ = 2.53, 95% CI: 1.89–3.40; OR_urban_ = 1.68, 95% CI: 1.55–1.82).

## 4 Discussion

This study presents a comprehensive national assessment of bicyclist injury hospitalizations in the United States from 2016 to 2020, with a focus on disparities between rural and urban settings. Across this period, bicyclist hospitalizations remained a consistent burden, with most cases occurring in urban areas but rural hospitalizations reflecting greater severity and resource use.

Over the 5‐year study period, 124,000 hospitalizations related to bicyclist injuries were estimated, with trends remaining relatively stable during the study period until a moderate increase in 2020. This rise may reflect pandemic‐related shifts in traffic behavior and increased bicycle usage. During lockdowns, bicycling activity surged, serving as both a substitute for public transportation and a form of recreation and exercise amid restrictions [22]. This change was accompanied by an increase of 8% in ED visits for nonfatal bicycle injuries compared with 2019 [23], a 3.6% rise in bicyclist fatalities compared with the same period over the previous 5 years [24], and on the basis of our findings, a 4.6% increase in inpatient hospitalizations compared with 2019, together pointing to an elevated burden on the healthcare system despite reduced motor vehicle traffic volumes.

The findings of our study also revealed marked disparities in injury patterns and hospital burden between rural and urban settings. Although most hospitalizations occurred in urban areas, likely because of greater population density and higher exposure rates, injuries in rural areas were slightly more severe. This pattern was evident across multiple measures: both the NISS and the RISS were marginally higher for rural patients, though not significantly so. Moreover, rural hospitalizations had a modestly longer LOS and higher total charges, suggesting a greater economic and clinical burden. These findings are consistent with prior research on other traffic users, reporting more severe injury outcomes for pedestrians in rural areas [25]. Similar patterns are evident among bicyclists. As an example, Boufous et al. [26] used police and hospital data in Australia and found that after adjusting for age, gender, helmet use, and crash type, bicyclists injured in rural areas had higher odds of sustaining severe injuries compared to those in urban areas (adjusted OR = 1.28, 95% CI: 1.00–1.71). Higher travel speeds, longer emergency response times, and fewer trauma‐designated facilities in rural areas were attributed to these elevated risks for vulnerable road users [26].

Rural and urban injury profiles diverged across multiple demographic and socioeconomic dimensions. Children and adolescents under 19 years of age accounted for a greater proportion of rural hospitalizations compared to urban hospitalizations, suggesting a greater burden on young bicyclists in rural areas. This aligns with Canadian data showing children aged 5–19 living in rural areas experienced notably higher hospitalization rates for bicycling injuries than their urban counterparts (urban 10.93 vs. rural 18.49 per 100,000). The authors further suggested that differences in hospital admission practices may contribute to these disparities, noting that rural hospitals, because of greater travel distances and limited access to diagnostic imaging and proper care facilities, may be more likely to admit injured children for observation even when injuries are less severe [11]. Furthermore, there was a pronounced increase in the likelihood of non‐routine discharges as age increased, a clear dose‐response pattern, particularly in rural settings likely due to the combination of older age and limited access to acute care.

There was also a reverse socioeconomic gradient observed; individuals from higher income quartiles were progressively less likely to be discharged non‐routinely compared with those in the lowest quartile. This pattern was observed among both rural and urban patients but was more pronounced among urban patients. In urban areas, lower‐income neighborhoods face disproportionately high pedestrian and bicyclist crash risk, with greater likelihood of severe or fatal outcomes [27]. These neighborhoods are often characterized by denser high‐risk road environments, including major arterials, multi‐leg intersections, and heavier motor traffic, which further elevate risk and severity [28]. Protective bicycling infrastructure substantially reduces injury risk (e.g., cycle tracks and separated facilities), yet infrastructure investments have historically been prioritized in more privileged neighborhoods, limiting safety gains where disadvantage is concentrated [29, 30].

In addition to age and income, racial and ethnic backgrounds were associated with discharge outcomes, though these patterns were less consistent. The odds of non‐routine discharge for Hispanic patients in urban areas were significantly lower than those for White patients. There have been mixed findings regarding the severity of injuries suffered by Hispanic bicyclists in localized studies. For example, an analysis of crash data from Los Angeles found that Hispanic bicyclists were less likely to sustain severe injuries [31]. In contrast, Raifman and Choma [32], using 2015–2019 data from the US Fatality Analysis Reporting System (FARS), reported disproportionately higher fatality rates per mile ridden among Hispanic bicyclists. The authors suggested that these disparities may reflect inequities in cycling infrastructure, differences in emergency response and medical care, and neighborhood‐level risks rather than individual behaviors [32]. These inconsistencies suggest that ethnic disparities in bicyclist injury outcomes are likely shaped by local factors, including roadway environment, safety infrastructure, and behavioral norms. Although prior studies have provided important insights within specific geographic areas, our findings offer a broader perspective on Hispanic injury outcomes in urban settings nationwide.

Another notable finding was the differing pattern of hospital discharges by sex. Males accounted for the majority of hospitalizations, yet in urban areas they were less likely than females to be discharged non‐routinely. This pattern was not observed in rural areas and in contrast with prior literature, male cyclists generally have higher probabilities of severe or fatal injury than female cyclists [33, 34]. One possible explanation might be the differences in cycling environments and perceptions of risk among urban male bicyclists. Prior research indicated that women tend to have a higher risk perception and feel less comfortable cycling in mixed traffic settings, and they tend to choose routes with less traffic [35]. Although intended to enhance safety, these preferences may paradoxically expose female cyclists to more hazardous conditions on sidewalks or shared‐use paths where vehicle interactions are more unpredictable [36]. Females may also have lower thresholds for hospital admission or discharge to post‐acute care due to clinical and social factors [37].

Beyond patient characteristics, crash‐related factors played an important role in discharge outcomes. Non‐routine discharge was significantly associated with motor vehicle involvement in both rural and urban settings, but the magnitude of the association was greater in rural areas. This is likely a result of rural environments that present inherently more hazardous conditions, where higher speeds, reduced visibility, and longer emergency response times contribute to the severity of crashes involving cyclists, which in turn increase the likelihood of non‐routine discharge. These findings are consistent with prior nationally representative research, which reported that bicyclists involved in MVCs had higher odds of non‐routine discharge compared with those involved in non‐MVCs (OR = 2.22; 95% CI: 2.06–2.39) [10]. Moreover, a larger proportion of rural patients were transferred, and a smaller proportion were admitted via the ED before hospitalization compared with urban patients, suggesting potential limitations in local trauma care. However, the difference in ED admission rates appears to be largely driven by the higher transfer rates among rural cases. After excluding transferred‐in cases, ED admission rates were comparable between rural and urban settings (84.1% vs. 89.9%, respectively). These findings highlight that rural bicycling injuries still place a higher clinical burden on less‐resourced health systems, as reflected by a greater need for inter‐facility transfers.

In terms of regional variations over time, the distribution of bicycling‐related hospitalizations shifted across some Census regions. Compared to estimates from 2002 to 2009, the South and Midwest maintained similar shares of hospitalizations, whereas the West increased from 29% to 34% and the Northeast declined from 25% to 20% [10]. These shifts likely reflect changes in cycling behaviors, infrastructure investments, and risk exposures across regions [38]. These findings align with broader national trends, as Western states such as California reported a nearly 2% increase in bicyclist fatalities in 2021, reflecting rising cycling activity and persistent safety challenges in urban and suburban corridors [39]. In terms of outcomes, urban patients in the Northeast and South had significantly higher odds of non‐routine discharges than those in the Midwest. On the other hand, rural patients in the West were less likely to undergo non‐routine discharges. The patterns may be attributed to differing infrastructures in rural and urban areas, behavioral differences between regions, and highlights the need for geographically tailored injury prevention and trauma care strategies.

This study has several limitations. This cross‐sectional analysis of hospitalization data provided an overall picture but did not establish causal relationships. Future research should employ longitudinal designs or data linkages to better understand causal mechanisms of injuries, recovery outcomes, and care access. The dataset lacks exposure information (e.g., cycling frequency or distance traveled), which prevents the calculation of injury risk estimates or comparison of rates across population subgroups. However, because this study focuses on post‐hospitalization outcome (e.g., discharge disposition), exposure is less relevant once an injury requiring hospitalization has occurred. Third, rurality was assigned on the basis of patient's residential location using NIS population‐based categories and dichotomized as urban (>50,000) versus rural (≤50,000). Because this residence‐based proxy does not reflect the exact crash location and is less granular than other classification methods such as the Rural–Urban Commuting Area (RUCA), which assigns rurality at the census tract or zip code level based on population density and commuting flows, some misclassification is possible. Additionally, unmeasured confounders such as helmet use and behavioral factors, crash dynamics, and pre‐hospital response characteristics (e.g., response time) were unavailable, all of which may influence both injury severity and discharge outcomes. Lastly, although discharge disposition serves as a meaningful proxy for clinical burden, it does not capture long‐term functional status or quality of recovery. Furthermore, the non‐routine discharge category included heterogeneous dispositions, such as death, transfers, and discharge against medical advice, which may reflect different clinical circumstances and underlying patient characteristics. Nevertheless, despite these limitations, our findings offer meaningful insights into the patterns and disparities of bicyclist injury hospitalization outcomes by rurality, helping to identify the risk profile of vulnerable road users and inform policy development and future research.

## 5 Conclusions

Bicyclist hospitalizations persisted and increased over the study period, a finding that underscores the importance of national‐level surveillance for characterizing the overall burden. Most injuries occurred in urban areas, yet rural cases showed slightly greater severity and hospital costs per patient, with disproportionate impacts on adolescents and children. Age was associated with discharge disposition in both settings, whereas other demographic factors (sex, race/ethnicity, income) mattered mainly in urban areas, which may reflect the relatively greater influence of environmental factors in rural settings (limited protective infrastructure, higher operating speeds, and fewer nearby trauma resources), thereby attenuating observable associations with demographic characteristics. Although most crashes were single‐bicycle incidents, motor‐vehicle involvement strongly predicted poor outcomes, especially in rural areas. Our findings highlight the need for equity‐focused interventions that address demographic disparities and rurality, such as community‐based education programs, and safe routes to school in rural areas, as well as equitable investment in protected bike lanes, safer intersections, and infrastructure improvements in underserved urban neighborhoods. Targeted measures to reduce motor vehicle–related crash risk and region‐specific strategies remain essential to guide both prevention and post‐acute care.

## Funding

This work was funded by the Association for the Advancement of Automotive Medicine (AAAM) through the 2023 H. Clay Gabler Scholar Award.

## Conflicts of Interest

The authors declare no conflicts of interest.

## Supporting information




**Table A1** Adjusted Odds Ratios (95% confidence intervals) for predictors of non‐routine discharge and their interactions with patient home (urban vs. rural).

## Data Availability

The data that support the findings of this study are available on request from the corresponding author. The data are not publicly available due to privacy or ethical restrictions.
