# Supplementary material for: National Burden of Bicyclist Hospitalizations in the United States by Rurality, 2016–2020
Source: J Rural Health. 2026 Jul 28;42(3):e70194. doi: 10.1111/jrh.70194 (PMC13411185; doi:10.1111/jrh.70194)
Supplement: Supplementary file 1 — Table A1 Adjusted Odds Ratios (95% confidence intervals) for predictors of non‐routine discharge and their interactions with patient home (urban vs. rural). [file JRH-42-0-s001.docx]

**APPENDIX:**

**Table A1:** Adjusted Odds Ratios (95% Confidence Intervals) for Predictors of Non-routine Discharge and Their Interactions with Patient Home (Urban vs. Rural)

|  | **Main Effects** | | **Interactions (Urban × Variable) ^1^** | |
| --- | --- | --- | --- | --- |
| **Variable** | **OR** | **%95 CI** | **OR** | **%95 CI** |
| **PATIENT CHARACTERISTICS** | | |  |  |
| 10-19 | REFERENCE | | | |
| 0-9 | 0.48 | 0.20-1.14 | 0.91 | 0.36–2.34 |
| 20–30 | 2.60 | 1.48-4.58 | 0.9 | 0.50–1.62 |
| 31-50 | 4.50 | 2.84-7.11 | 0.73 | 0.45–1.18 |
| 51–70 | 7.34 | 4.74-11.39 | 0.88 | 0.56–1.40 |
| ≥71 | 15.64 | 9.48-25.8 | 0.78 | 0.46–1.32 |
| **Sex** |  |  |  |  |
| Female | REFERENCE | | | |
| Male | 1.10 | 0.87-1.44 | 0.74 | 0.57–0.96 |
| **Race / ethnicity** |  |  |  |  |
| White | REFERENCE | | | |
| Black | 0.91 | 0.57-1.45 | 1.11 | 0.68–1.80 |
| Hispanic | 0.70 | 0.38 – 1.31 | 1.18 | 0.62–2.22 |
| Other | 1.14 | 0.66 – 1.99 | 0.79 | 0.45–1.40 |
| **Primary Insurance Payer^*^** | | |  |  |
| Self-pay | REFERENCE | | | |
| Medicare/Medicaid | 3.23 | 2.04–5.13 | 0.82 | 0.51–1.34 |
| Private Insurance | 1.84 | 1.14–2.95 | 0.92 | 0.56–1.52 |
| Other/Unknown | 1.99 | 0.98–4.05 | 0.77 | 0.37–1.62 |
| **Median Household Income** | |  |  |  |
| Quartile 1 (Lowest) | REFERENCE | | | |
| Quartile 2 | 1.11 | 0.87–1.43 | 0.9 | 0.69–1.17 |
| Quartile 3 | 1.05 | 0.75–1.47 | 0.88 | 0.62–1.25 |
| Quartile 4 (Highest) | 0.88 | 0.49–1.58 | 1.0 | 0.55–1.80 |
| **Hospital Region^*^** | |  |  |  |
| Midwest | REFERENCE | | | |
| Northeast | 1.43 | 0.98–2.07 | 0.91 | 0.62–1.34 |
| South | 0.97 | 0.72–1.30 | 1.17 | 0.86–1.61 |
| West | 0.63 | 0.47–0.86 | 1.63 | 1.18–2.24 |
| **Admission Day** | |  |  |  |
| Weekend | REFERENCE | | | |
| Weekday | 1.22 | 0.96–1.55 | 0.82 | 0.64–1.06 |
| **Transfer Status** | |  |  |  |
| Not transferred | REFERENCE | | | |
| Transferred in | 1.17 | 0.92–1.49 | 0.93 | 0.72–1.21 |
| **ED Admission** |  |  |  |  |
| No | REFERENCE | | | |
| Yes | 0.97 | 0.73-1.29 | 0.88 | 0.65–1.20 |
| **Motor Vehicle Involvement (MVC)** | | |  |  |
| Non-MVC | REFERENCE | | | |
| MVC involvement | 2.53 | 1.89–3.40 | 0.67 | 0.49–0.90 |

^1^ All interactions are relative to the Rural × reference category combination.
